# Plant-Based Meat Analogues and Consumer Interest in 3D-Printed Products: A Mini-Review

**DOI:** 10.3390/foods13152314

**Published:** 2024-07-23

**Authors:** Owen Miller, Christopher J. Scarlett, Taiwo O. Akanbi

**Affiliations:** School of Environmental and Life Sciences, College of Engineering, Science and Environment, The University of Newcastle (UON), Brush Road, Ourimbah, NSW 2258, Australia; owen.miller@newcastle.edu.au (O.M.); c.scarlett@newcastle.edu.au (C.J.S.)

**Keywords:** plant-based meat analogue (PBMA), 3D printing, plant protein, consumer acceptance

## Abstract

The markets for plant-based meat analogues (PBMAs) are growing worldwide, showing the increasing consumer demand for and acceptance of these new products. Three-dimensional (3D) food printing is a new technology with huge potential for printing products customised to suit consumers’ wants and needs. There is a broad acceptance from consumers regarding the safety and desirability of consuming food products that are produced using 3D printing. As this is a new technology, consumers must be provided with relevant information from a trusted source, with further research needing to be conducted within the context of the identified market and culture. By embracing the strength of customisation of 3D printing and coupling this with the global demand for plant-based products, 3D printed PBMAs could be a future challenger to the currently popular production method of extrusion. Therefore, this article reviews consumer interests in PBMAs and summarises opportunities for using 3D printing technology to produce plant-based meat analogues.

## 1. Introduction

The predicted population growth, with its concomitant increase in food demand, will have an impact on global food markets [1]. Recent years have seen rapid growth in plant-based meat analogues (PBMAs) [2], with more than 6485 new products launched globally from 2015 to 2021 [3]. Consumers have increased their interest in plant proteins, which have been promoted as a healthier lifestyle choice and led to increases in the trends of flexitarianism, vegetarianism and veganism [4,5,6,7,8]. A recent survey in 2023 has revealed that almost 2 in 5 (39%) Australians are actively trying to reduce their meat consumption, and slightly fewer Australians (38%) are open to substituting meat products with plant-based alternatives [9].

The other driving factors behind this shift have been the growing concerns regarding sustainability and animal welfare issues, such as the impact of the meat industry on the environment, deforestation and pollution [6,7,10,11,12]. This has led to the promotion of PBMAs as “better for you” and “better for the planet” compared to animal products [3]. The increase in demand for non-animal protein sources is also predicted to be impacted by the massive market expansion for halal and kosher food products [13].

Three-dimensional (3D) food printing is a relatively new technology being explored in the food industry, which can build a three-dimensional object layer-by-layer based on a programmed path [14]. At present, extrusion-based 3D printing is the most popular over the other types of 3D printing: selective laser sintering, binder injection, and inkjet printing [15,16]. While this technology is becoming increasingly popular for producing chocolate and other confectionary products [17], research into the printability of various food inks has been expanding. These include soft meat products, cheese, pizza dough, fish and seafood [18].

Currently, the vast majority of commercial PBMA products are produced through the process of extrusion [16]. 3D printing, also called additive or extrusion printing, is a new technology being explored using plant proteins. It has demonstrated the ability to produce PBMAs (Table 1) and offers many advantages over the process of extrusion.

This mini-review will explore the consumer interest in PBMAs and the potential of using 3D printing to produce PBMAs. It will also investigate whether there will be acceptance by consumers of the use of this new technology for producing food products. Specifically, the following three areas will be explored:

1. The current state of the PBMA global market and consumer interest in PBMA commercial products.
2. Three-dimensional (3D) food printing and consumer acceptance of food products using this new technology.
3. Advantages and opportunities for 3D printing using plant proteins.

## 2. Plant-Based Meat Analogue Markets and Consumer Interest

The global PBMA market was estimated to be USD 1.6 billion in 2019, with a projected annual growth rate of 12% [3]. Predictions for the future market value of PBMAs show considerable variation. However, they all show a large amount of growth from the current market valuation (Table 2). This has seen a rapid increase in investment in food technology, and as the value of the opportunity rises, the technology providers are also increasing their research into the alternative protein space [24]. International estimates on the current state of the market have estimated that Europe holds the largest share (51.5%), followed by North America (26.8%), Asia Pacific (11.8%), Latin America (6.3%) and Middle East and Africa (3.6%) [3]. In the PBMA market space, the top 10 companies are Beyond Meat, followed by Boulder Brands, Hain Celestia, Nestle, Garden Protein International, Vivera, Lightlife Foods, Woolworths, Naturli Foods and Sainsbury’s [3].

This opportunity for expansion is being contested by many established businesses and recent startups, trying to differentiate their products and solve emerging technological issues [24]. Solving these issues will likely yield significant intellectual property for these innovators. In the context of current market predictions, the value of this intellectual property could be measured in hundreds of millions and even billions of dollars [24]. Research and innovation are needed in production methods for commercial products and throughout the entire supply chain, including the supply, processing, and formulation of new PBMAs.

Given the current state of the market, it has been concluded that there is a high demand for PBMAs, now and in the future, due to what has been termed emerging consumer drivers, which are centred around healthy diets and environmental sustainability [6,16,31,32,33,34,35,36,37].

The main barriers for consumers to purchase PBMAs have been identified as unfamiliarity and a lower sensorial experience [38,39,40]. The additional barriers of price and convenience have also been noted, though they appear to be given less weight by consumers [41]. Once the barrier of unfamiliarity has been overcome, regular consumers of PBMAs tend to rate them as better than meat; in contrast, non-consumers of PBMAs tend to rate meat as much better [38]. This has been supported by another study into consumer behaviour, which found that those familiar with meat were less attracted to meat alternatives [42].

Consumers’ reasons for consuming PBMA have also been shown to vary across countries [43]. The attitudinal predictors of consumers in the USA for consumption of PBMA were excitement, appeal, and low disgust. Whereas in China, they were healthiness, tastiness, and sustainability; in India, it was excitement, necessity, and goodness [43].

The barrier of sensorial experience can be further broken down into its components of flavour, texture, colour, taste, and overall appearance [3,24,44]. The convenience of using PBMAs involves their cooking performance, fit to culinary traditions and nutrition [24]. Consumers’ acceptance of PBMAs is also affected by several motivational barriers, including food neophobia, social norms and rituals, and consumers’ eating goals [45]. This has been summarised as making PBMAs attractive to average meat-eating consumers rather than primarily to vegetarians and vegans. PBMAs can be considered as a product that can help consumers reduce their meat consumption through the substitution of meat [46]. This segment of the population has been identified as flexitarians and is crucial to the rise in the PBMA market [5]. This has led to the industry tackling the barriers to the desirability of consuming PBMA by formulating products that have meat-like flavours, textures, appearance, and functionality in traditional meals.

It has been found that consumers did not assume that PBMAs were more sustainable and healthier just because they were meat-free [47]. It is possible that consumers underestimated the environmental impact of animal-based products and overestimated the impact of PBMAs. Consumers also evaluated PBMAs as less ‘natural’ than traditional meat products, which led them to assume that PBMAs are less healthy [47]. Consumers perceive food processing and additives as negative [48], with long ingredient lists as less natural [49]. This highlights a major challenge for the acceptance of PBMAs by consumers, as they are classed as ultra-processed food, which can give them an undeserved automatic unhealthy image [16]. When considering the healthiness of commercial PBMAs, in terms of nutritional quality at a macro scale, there has been found to be an equivalence of PBMAs with meat products; however, on a microscale, many PBMAs lack important vitamins and minerals [16]. Other studies have found that consumers perceive eating less meat to be an ineffective action they can take to reduce their environmental impact [50,51]. This consumer view of reducing meat intake as being ineffective could be linked to their low willingness to stop eating meat when they were provided with a list of pro-environmental actions [50]. This highlights the importance of providing information to consumers on the higher sustainability of PBMAs compared to traditional meat products [47], which is becoming an important consideration for dietary guidelines from numerous countries [16,52]. It also reinforces that PBMAs should be formulated to be healthier than the meat products they mimic to remove possible barriers to the acceptance of PBMAs.

Promoting the positive aspects of PBMAs could lower the barriers for consumers [53]. By providing information that shows that PBMAs are more sustainable with lower environmental impacts, as well as positive impacts on animal welfare, compared to traditional meat products, it has been proposed that consumers’ intent to purchase PBMAs would increase [53]. This strategy of providing information on PBMAs has been shown to be effective in improving consumer acceptance [54]. Changing the perception of consumers could motivate them to seek out non-animal-based food options [35].

## 3. Three-Dimensional (3D) Printing and Consumer Acceptance

One of the most recent innovative food production methods is 3D printing, and the demand for this emerging technology is increasing. It is an additive manufacturing technology that has numerous advantages. While the uptake of this technology continues to grow, consumer acceptance of food produced this way is mixed. The 3D food printing technology and consumer acceptance of the technology are explored below.

### 3.1. 3D Food Printing Technology

**Figure 1:**
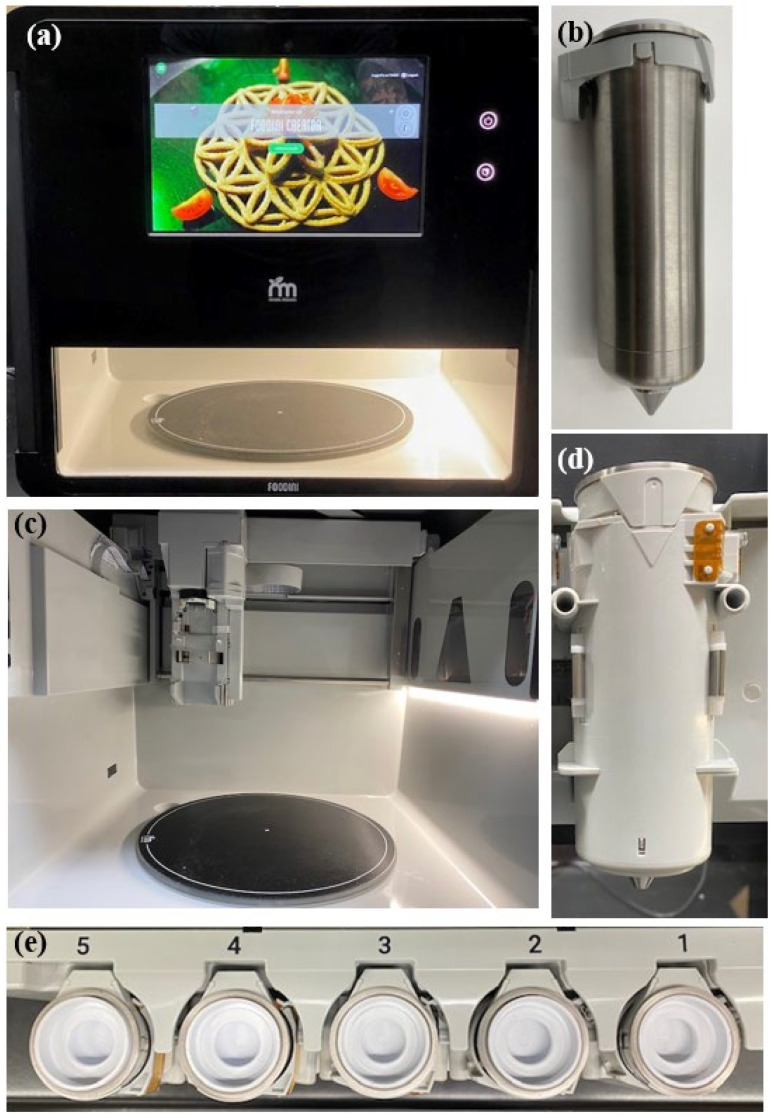
A commercial extrusion food printer showing: (**a**) Front view of a commercial food printer; (**b**) Front view of an ejected single food canister to be loaded with food ink; (**c**) Internal printing stage; (**d**) Front view of a loaded single food ink canister; (**e**) Top view of five food ink canisters labelled to correspond with printing software.

3D food printers (Figure 1) can be used to build a three-dimensional object layer-by-layer based on a programmed path [14]. This process includes three steps: feeding, slicing, and printing [55]. Feeding is the process of adding the raw plant protein material in a paste or molten form with suitable rheological properties into the 3D printer. Slicing is whereby the 3D printing software processes the 3D-designed model into slices to be printed. The final step of printing is where the raw materials are extruded from the printing nozzle and deposited onto a platform to form the designed product [56]. 3D printing has potential for the PBMA market although it is only at a laboratory-based stage [55,56]. There are some recent startups, however, aiming to use this new technology to produce commercial products [57].

One of the biggest obstacles to the use of 3D printing to produce PBMAs has been balancing the problem of fluidity of the raw materials. If the fluidity is too low, then the nozzles can quickly become blocked; if it is too high, then the product can deform on the printing platform [55]. This has led to investigations into additives, such as hydrocolloids [19], which may be used to improve the structural integrity and the texturization of a printed product when printing with plant proteins without compromising the flow of the food ink. Consequently, additives are being investigated that can allow for a food ink to have an appropriate fluidity and allow the printed product to have a stable shape without deformations (minor or major), as this can affect the overall perceived quality of the printed product.

The major advantage of 3D printing is that it can be easily customised in terms of the nutritional components, flavour, colour, shape and texture of the final product [16,56]. One example of customisation is that the size of the fibres and/or layers can be determined by the nozzle aperture of the 3D printer. Therefore a smaller nozzle can be used to build a 3D model on a microscale which could mimic the structure of an animal’s muscular tissue [56]. With regards to nutrition, 3D printing could allow for the incorporation of varied levels of macro- and micronutrients based on a required nutritional profile for different populations within different products from a single 3D printer. The sensory quality of a printed product can be evaluated from customised formulations to ensure that it will match consumer preferences in terms of desired organoleptic properties including texture, mouthfeel, taste and flavour. For example, elderly populations may require a different nutritional and textural profile compared to children [56]. 3D printers with multiple printer heads can simultaneously print different materials which allows for an even higher degree of nutrition customisation from a single 3D printer.

### 3.2. Consumer Acceptance of 3D Printed Foods

One of the biggest challenges with consumer acceptance of 3D food printing has been the unfamiliarity of consumers with the new technology [58]. However, consumers have been found to have a higher acceptance of disruptive new food innovation technologies when they have been developed by small producers compared to large corporations [59]. Smaller producers are able to emphasize the naturalness and the positive effects that the new technology can have on health, the environment, and society. A study into consumer acceptance of 3D-printed food production found that consumers were more likely to purchase 3D-printed meatballs instead of conventional meatballs, which demonstrated an acceptance of the technology regarding food production [60]. The participants in the study also thought the 3D printed products (cookies, meatballs, and pizza) were less processed than the conventional products. However, a contrasting study found that consumers had doubts about the healthiness of printed foods and were concerned with their unusual appearance [61].

Producing food products using 3D printing should focus on creating an appearance that is acceptable and familiar to consumers to overcome their neophobia [61]. There is also a need to be transparent in regard to their nutrition, production method, and freshness to increase consumer acceptance [61,62]. In a recent survey on consumer attitudes towards 3D food printing, only 91 out of 329 participants (28%) viewed it as unacceptable, while 140 participants (42%) were excited to try, eat and buy 3D printed food products [60]. The remaining 91 participants were moderately interested in 3D printed products, thinking 3D printed products were somewhat safe yet they were still excited to try 3D printed food.

A recent study has shown that providing information on 3D food printing can improve its acceptance by consumers [58]. Initially, consumers gave little credit to 3D-printed foods, and they had a negative attitude, which was linked to neophobia. After the provision of information through a written questionnaire there was an improvement in attitudes [58]. Namely when the arguments concerned the fun, convenience, health and personalised nutrition aspects of 3D printing and how it could add value for them. It was noted that the factual presentation of this information did not appeal to all the participants and that future marketing and research should investigate alternative communication forms. Exposing consumers to positive experiences with 3D-printed food products is another method which can lead to improved acceptance [62]. It has been suggested that it is highly important that an early and well-designed information campaign be undertaken to control and anticipate communication about a new technology [58]. Also, food producers should seek precise information consumers would like to have available on food packaging and knowledge on whose validation consumers would trust, as this would be highly valuable [63].

## 4. 3D Printing Technology and the Future of PBMA Production

As the study into using 3D printing to produce PBMAs is relatively new, many opportunities exist for further investigation. The use of a greater number of protein sources, singular and blended, and nutrient-rich additives that can demonstrate the nutritional potential of 3D-printed PBMAs is only beginning to be explored. This will involve investigations into the rheological properties of new mixtures, including the impact of water-to-protein ratios, the chemistry of the protein sources and their interactions with other additives. These studies can start to explore the design space for 3D printed PBMAs, which is intimidatingly vast due to the plethora of ingredient combinations and the complexity of processing parameters.

### 4.1. Customisation

The advantage that 3D printing PBMAs, or any food product, should endeavour to maximize is customisation. Customisation can involve almost every aspect of the PBMA that is being printed, including size, shape, sensorial experience (such as colour and flavour), nutritional goal, allergenicity concerns, and fortification with vitamins and minerals [16,56]. Allowing consumers control over these qualities of the PBMA is a level of transparency and choice that they are not afforded with other manufactured food products. This would come with a high cost regarding the systems needed to attain and process this information, as well as provide feedback to consumers about the viability of their choices. However, if the benefits to the consumer are communicated and the design process is easy and intuitive, this would be an extremely valuable asset for 3D-printed PBMAs, which should be embraced.

### 4.2. Production

3D printing can challenge the current model of central production and distribution of PBMAs. 3D printers do not require large industrial manufacturing plants, as the machines can be relatively small and only take up a minimum of bench space for operation. This portability of 3D printers allows the 3D printer itself to be moved to a location of demand, which means that production can be on-site for the consumer. This could have positive benefits regarding the shelf life and stability of the product, and improve the perceived ‘freshness’ of the product by consumers if they know the actual site of production is local.

### 4.3. Ingredients

Future research will need to focus on the printability of different plant proteins and which additives and combinations of raw materials can be used to build PBMAs with desirable sensorial properties [64,65]. Different protein pastes will have different viscosities and viscoelasticity, where having appropriate values for these two properties is vital for printability [56]. Edible gums have been investigated as flow enhancers, and hydrocolloids can improve shape stability and decrease the clogging of nozzle tips [19].

3D printing can give consumers control over the ingredients that are used in PBMA formulations. This can be important for consumers to control the presence of known allergens or different nutritional goals. Some ingredients are commonly used in commercial PBMAs which are known allergens, namely soy, pea and gluten [16,66,67,68]. 3D printing can allow consumers to ensure that any ingredients that they are allergic to are not included in their customised printed PBMA. Consumers may also have different nutritional goals and will therefore have different macro-nutrient requirements to reach those goals. Allowing consumers to customize the formulation for a PBMA can allow them to adjust the amounts of fibre, salt, protein, carbohydrate, and fat. However, the ease of incorporating these choices into commercially 3D-printed PBMAs is likely not an easy barrier to overcome.

Another area of growing interest to researchers and food manufacturers is the production and usage of a broad range of functional ingredients [69,70], which can then be utilised in the development of PBMAs. New technologies that produce individual ingredients, such as precision fermentation, are growing rapidly. The process of precision fermentation has been predicted as a sustainable method to produce ingredients for future PBMAs [24]. For example, precision fermentation is currently used to produce soy leghemoglobin, which is used by Impossible Foods to create the flavour and colour of meat in their PBMAs [71]. There is also the potential for precision fermentation to produce fats and oils more sustainably, and consumers could see this method as more ethical than the use of animal products. The inclusion of these lipids could have a positive impact on the sensory quality of PBMAs through improvements in flavour and mouthfeel. For example, Nourish in Australia has recently produced animal-free fats that can be used to give products the flavour of traditional meat without animal ingredients [72].

The use of transglutaminase in 3D printing of protein-rich products has been explored. This enzyme, when mixed with a protein mixture before printing, can form cross-links between proteins, leading to changes in rheological properties like hardness and cohesion [73]. Importantly, this can be achieved without the high temperatures and pressures typically associated with high- and low-moisture extrusion processes. However, the printing process itself can be intricate and may result in nozzle clogging [56].

### 4.4. Structure

**Figure 2:**
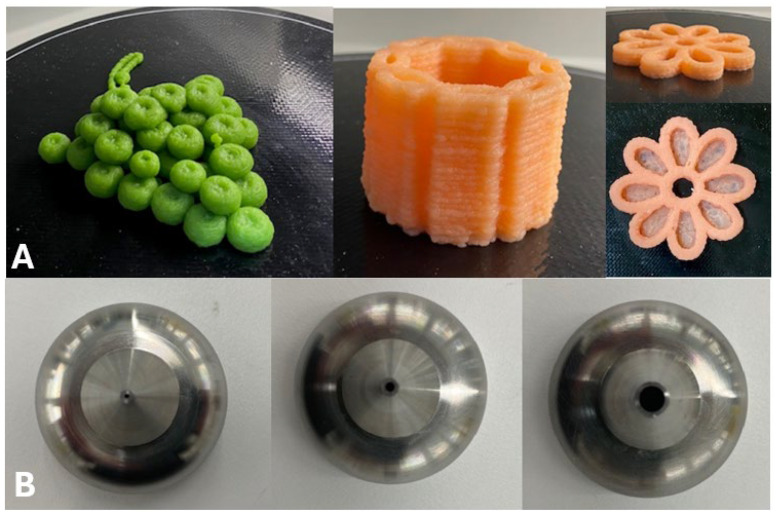
Demonstration of the customisation potential of 3D printing to produce a variety of appearances. (**A**) Designs printed from a commercial 3D printer using a formulation of commercially available packet-mix mashed potato and water with different food dyes added, (**B**) Available nozzle sizes for a commercial 3D food printer: 0.8 mm, 1.5 mm, and 4.0 mm (left-to-right).

Customisation of the structure is a new area of research which has only begun to be explored. Changes in the printed structure of a PBMA have the potential to have significant impacts on the sensory properties of the product, the stability of ingredients used in formulation, increasing consumer choices, varying serving sizes, and assisting consumers with dysphagia. The effect of infill pattern and infill ratio on the texture of 3D printed PBMAs has begun to be investigated [19]. Further study into the interaction of these properties of a 3D model with proteins and lipids used in PBMA formulations makes this a vast landscape for exploration. Customisation of the structure of printed PBMAs could allow consumers to choose from a variety of available appearances using the same formulation (Figure 2A). Figure 2A shows a variety of structures that were printed from the printer in Figure 1 using a mashed potato packet mix and water with the addition of food dye. Changing the internal structures of PBMAs during printing can also vary their textural properties [19], such as hardness and chewiness, and increase consumers’ satisfaction with printed products. This ability to vary the texture and appearance of a printed product can also improve the appeal of food for patients with dysphagia, which can improve their energy and protein intake [74]. Currently moulded food is typically used for patients with dysphagia which can require high levels of starch to retain its shape, which results in a gelatinous mouth feel [74].

A single 3D printer can have the capacity to print multiple inks through different-sized nozzles (Figure 2B). This further increases the design space for printed food products as it can allow for a higher degree of complexity of printed models. This could include enclosing a food ink within a larger structure, such as enclosing lipids within a protein structure to reduce possible oxidation and increase stability. Also, printing fine details on the external faces of a 3D model, such as writing, is another customisation aspect that may affect the appearance of the printed product and improve its appeal to consumers.

Another method of varying the structure is the use of a coaxial nozzle which allows one material to be extruded within a second material in which it is encased. A coaxial nozzle has been used to produce a plant protein-wrapped fibre solution that could mimic traditional meat [75]. A coaxial nozzle could also be used to encapsulate nutrients and flavour compounds from environmental influences during processing and packaging, which could extend shelf-life and improve the stability of the PBMA [56,76].

### 4.5. Four-Dimensional (4D) and Five-Dimensional (5D) Printing

**Figure 3:**
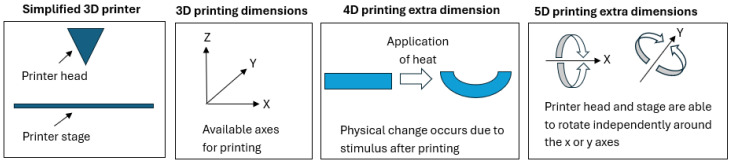
A comparison of the printing dimensions for 3D, 4D and 5D printing.

4D printing is the next step that is being explored from 3D printing (Figure 3). 4D printing involves a physical or chemical change of state through time when the 3D printed object reacts to environmental stimuli or human intervention [77]. The rheology of food inks which can function in the capacity of 4D printing is beginning to be explored using a variety of formulations [78,79,80,81,82]. 4D printing includes colour changes, such as when a food ink changes colour after printing due to exposure to a change in pH [78] or due to exposure to heating via microwaves [81].

5D printing is another advance where the printer head and the movement of the printer bed/base at a variety of angles allow for the printing of curved rather than flat layers [77]. This allows for products to be built from bottom to top, layer by layer along a vertical axis, as it adds the two axes of movement of the printer head and the printer bed (Figure 3), which can allow for layers to be stacked on a vertically tilted axis allowing for curved surfaces and more complex structures to be printed [77]. Printing food products this way would require detailed knowledge of the rheological properties of food inks. In the case of PBMAs, there must be a proper understanding of the flow properties of ingredients prior to printing. 5D printing could allow for extreme levels of accuracy and precision and a high degree of complexity of the food matrix from printed products [77]. The cost would be much higher than 3D printing due to the required equipment and knowledge. However, 5D printing has the advantage of a much higher potential for customisation and the ability to satisfy consumer desires.

## 5. Conclusions

To improve consumer acceptance of PBMAs, providing information on their health and environmental benefits could be a pivotal component of new marketing strategies. Due to the unfamiliarity of 3D printing for consumers, acceptance and trust are highly important to build. This can be achieved through transparency in all aspects of the design and creation of these new products.

Using new technologies, such as 3D printing, can offer many advantages but there will need to be considerable investment in educating consumers on the safety and benefits of this new mode of food production to ensure consumer acceptance. The advantages that 3D printing offers over current methods of PBMA production do not match up to the conventional food production model of centralisation of production. To embrace these advantages to their full potential would need a more decentralised model to produce customised products rather than a mass production model where one size fits all.

One of the major strengths of 3D printing PBMAs is the ability for consumers to customize almost all aspects of the design process. This ability to customize will be important for the 3D printing of PBMAs to gain a competitive advantage against current commercial PBMA products. This can be undertaken through the use of technology to assist consumers in the design process of choosing their ingredients and the textural properties they desire in a PBMA.

While 3D-printed commercial products are still not widely available, there has been increasing research into the viability and safety of a variety of food inks. Awareness of the advantages of this technology for consumers will need to be raised and all safety concerns addressed for 3D printed products to be commercially successful.

## Figures and Tables

**Table 1:** Plant proteins which have been used in 3D-printed PBMAs and the purposes for using additives to improve the quality of the printed product.

Plant Protein(s)	Additive	Purpose for Addition	References
Soy	Hydrocolloids	Effect on textural properties	[19]
Pea	Wheat bran	Nutritional enrichment	[20]
Soy and Pea	Mushrooms	Nutritional fortification	[21]
Mung Bean	Xylose	Improved structural properties	[22]
Mung Bean	Transglutaminase	Effect on textural properties	[23]

**Table 2:** Predictions of the global market value for plant-based meat analogues.

Prediction Year	Predicted Value of Market	References
2025	USD 21.23 billion	[25]
2026	USD 30.9 billion	[26]
2027	USD 15.7 billion	[27]
2028	USD 16.78 billion	[28]
2030	USD 24.80 billion	[29]
2032	USD 30.60 billion	[30]

## Data Availability

No new data were created or analyzed in this study. Data sharing is not applicable to this article.
